# Normalization of Reference Ranges for Laboratory Tests Using a Novel Rescaling Approach for Optimal Clinical Interpretation

**DOI:** 10.7759/cureus.84230

**Published:** 2025-05-16

**Authors:** Dhayananth Rattaipalivalasu Saravanan

**Affiliations:** 1 Internal Medicine, Mercy Health St. Vincent Medical Center, Toledo, USA

**Keywords:** ai and machine learning, applications of medical informatics, data standardization, electronic clinical decision support system, electronic health records (ehr), laboratory interpretation, laboratory normalization, reference range, rescaling method, standardized lab values

## Abstract

Laboratory result interpretation is often hindered by variability in reference ranges across different institutions and analytes. This inconsistency can delay clinical decisions and increase cognitive effort. We present a normalization method that transforms laboratory values into dimensionless scores using simple rescaling formulas based on the reference range. By converting values to a 0 to 1 or -1 to 1 scale, this approach allows intuitive assessment and direct comparison across different tests, regardless of their original units. It simplifies recognition of abnormal values, enhances clinical efficiency, and is suitable for integration into electronic health records and algorithmic tools. Applied to common laboratory tests, the method demonstrated improved interpretability and clarity. Normalizing lab values in this way offers a practical, scalable solution to support faster and more accurate clinical decision-making.

## Introduction

Laboratory tests are integral to medical decision-making, yet their interpretation remains a complex task due to the variability in reference ranges. Traditionally, reference intervals are established based on the central 95% of values from a presumed healthy population, but these intervals can vary significantly between laboratories owing to differences in instrumentation, population demographics, and assay calibration techniques [1,2]. Such variability challenges clinicians, who must quickly recognize abnormalities in a sea of numeric data, often under time-sensitive conditions.

Compounding this difficulty is the fact that identical numeric deviations across analytes do not carry equal clinical weight. For instance, a 2 mEq/L drop in sodium may warrant observation, whereas a similar drop in potassium could be life-threatening [3]. Despite the critical nature of this contextual interpretation, current systems provide minimal support for intuitive cross-analyte comparison.

Previous efforts to improve interpretability have focused on refining reference ranges or subgroup stratification by age, sex, and other factors [4]. However, these approaches do not solve the core challenge: the lack of a unified, dimensionless framework for comparing test results across different analytes.

In this paper, we propose a normalization technique that transforms raw laboratory values into standardized, unitless scores based on their respective reference intervals. By rescaling results to either a 0-1 or -1 to 1 range, this method facilitates rapid interpretation and supports consistent evaluation across diverse laboratory parameters. Such normalization not only benefits bedside clinical decision-making but also provides structured input well-suited for integration with machine learning algorithms and electronic health record (EHR) systems [5].

## Technical report

Materials and methods

To facilitate consistent interpretation of laboratory results, we applied two normalization formulas that rescale test values relative to their reference ranges. These formulas convert values into dimensionless scores, allowing intuitive comparison across analytes with differing units or thresholds. The concept of transforming results based on reference intervals has been recommended for decades to support clinical utility and reduce misinterpretation due to inter-laboratory variation [1-3].

The two formulas are as follows:

(1) Normalization to a range of 0 to 1:

\[ x' = \frac{x - x_{\text{min}}}{x_{\text{max}} - x_{\text{min}}} \]

(2) Normalization to a range of -1 to 1:

\[ x' = \frac{2(x - x_{\text{min}})}{x_{\text{max}} - x_{\text{min}}} - 1 \]

Where:

$x$: actual lab value

$x_{\text{min}}$: lower bound of the reference range $x_{\text{max}}$: upper bound of the reference range $x'$: normalized (rescaled) value

These formulas were applied to commonly ordered lab tests (e.g., sodium, potassium, hemoglobin, creatinine, glucose) for demonstration. Values exceeding the upper reference limit yield results >1.0; those below the lower limit yield values <0 (for the -1 to 1 scale) or 0 (for the 0-1 scale). These transformations are well-suited for integration into EHR-based decision support tools and machine learning systems [4,5].

Results

Normalized scores provided consistent, interpretable results across tests. Application of both normalization formulas yielded interpretable, scalable results across multiple tests. These transformations highlight the degree of deviation from the reference range, facilitating rapid clinical prioritization and supporting standardized input for downstream modeling and analytics [5]. Tables 1-2 illustrate the application of the 0 to 1 normalization method on five routine laboratory parameters. This transformation contextualizes the severity of deviation without requiring familiarity with specific units or reference cutoffs. For demonstration purposes, representative values for common laboratory tests (sodium, potassium, hemoglobin, creatinine, and glucose) were selected based on standard clinical scenarios; these were not derived from patient records but used solely to illustrate the normalization method.

**Table 1:** Normalized laboratory values (0 to 1 scale). *Values >1.0 indicate results exceeding the upper reference limit.

Test	Reference range	Patient value	Normalized value	Clinical interpretation
Sodium	135-145 mEq/L	133	0.20	Mild hyponatremia, no urgent intervention
Potassium	3.5-5.0 mEq/L	1.5	0.00	Severe hypokalemia, urgent intervention
Hemoglobin	13.5-17.5 g/dL	11.0	0.25	Moderate anemia, further evaluation needed
Creatinine	0.6-1.2 mg/dL	2.4	1.00	Severe renal dysfunction, urgent consultation
Glucose	70-99 mg/dL	250	2.00*	Severe hyperglycemia, urgent management

**Table 2:** Normalized laboratory values (−1 to 1 scale). *Values >1.0 or <−1.0 indicate measurements beyond the reference boundaries.

Test	Reference range	Patient value	Normalized value	Clinical interpretation
Sodium	135-145 mEq/L	133	-0.60	Mild hyponatremia, no urgent intervention
Potassium	3.5-5.0 mEq/L	1.5	-1.00	Severe hypokalemia, urgent intervention
Hemoglobin	13.5-17.5 g/dL	11.0	-0.50	Moderate anemia, further evaluation needed
Creatinine	0.6-1.2 mg/dL	2.4	1.00	Severe renal impairment, urgent consultation
Glucose	70-99 mg/dL	250	3.00*	Severe hyperglycemia, urgent management

## Discussion

The normalization of laboratory test results into a standardized numerical scale addresses a longstanding barrier in clinical medicine: the variability and fragmentation inherent in traditional reference intervals. Rather than depending on disparate units and ranges, this approach provides a consistent framework for comparing physiological deviations across analytes. This is particularly advantageous in situations where clinicians must rapidly assess multiple lab values under pressure [1].

The key benefit lies in cognitive simplification. By converting diverse results into a single scale, the normalization process reduces mental load, allowing clinicians to interpret complex panels more efficiently. This aligns with calls for improved decision support tools that enhance clarity without sacrificing clinical nuance [2]. Unlike conventional alerts that flag values as *high* or *low*, normalized outputs convey the magnitude of deviation, which can be crucial for triaging urgency, especially in abnormalities that fall just outside traditional reference thresholds but are clinically significant.

This methodology also offers substantial utility for computational applications. Machine learning models, clinical decision support systems, and triage algorithms typically require standardized input formats. The use of normalized values as features aligns with best practices in data science, improving algorithm interpretability and potentially enhancing model performance [3,4].

Furthermore, normalized values enable consistent thresholds across institutions and patient populations, offering a step toward harmonization in laboratory data usage, a goal emphasized by international consensus efforts [5]. However, the method is not without limitations. It assumes linearity within reference ranges and may not account for biological variability at the extremes. Additionally, further work is needed to validate its clinical impact through prospective studies and EHR-based implementation trials.

Nonetheless, this rescaling strategy provides a scalable, interpretable, and integration-friendly enhancement to laboratory test interpretation, bridging the gap between raw data and clinical action.

## Conclusions

Transforming laboratory test results into normalized, dimensionless scores offers a practical solution to the interpretive challenges posed by varied reference intervals and units. By enabling direct, intuitive comparison across analytes, this approach has the potential to streamline clinical workflows, reduce cognitive burden, and support data-driven decision-making. Its compatibility with electronic health record systems and machine learning frameworks further enhances its applicability in modern healthcare. Future studies should evaluate real-world integration, clinician usability, and impact on diagnostic efficiency and patient outcomes.
